# Liposarcoma of the Nasopharynx: Diagnosis and Management of a Rare Diagnostic Entity

**DOI:** 10.1155/2012/314697

**Published:** 2012-03-26

**Authors:** George X. Papacharalampous, Dimitrios Kikidis, Alexios Vasileiou, Aggeliki Bousiotou, Aristeidis Chrysovergis

**Affiliations:** ^1^1st Department of Otolaryngology Head and Neck Surgery, Hippokration General Hospital, School of Medicine, National and Kapodistrian Unversity of Athens, 114 Vas. Sophias Avnue, 11527 Athens, Greece; ^2^Department of Pathology, Hipporation General Hospital, 114 Vas. Sophias Avnue, 11527 Athens, Greece

## Abstract

Liposarcoma is one of the most frequently occurring soft tissue sarcomas in adulthood. The majority of liposarcomas arise in the lower extremities and retroperitoneum, while the incidence of this tumor in the head and neck region is reported to be extremely low, comprising 1.8%–6.2% of all cases. Nasopharyngeal liposarcoma is exceptionally rare, with only three cases having been reported in the English literature. This paper presents a case of a nasopharyngeal liposarcoma, treated with endoscopic tumor debulking, followed by adjuvant chemotherapy and radiotherapy, and reviews the current literature with regard to diagnosis and management of such lesions. Most authors agree that the imaging modality of choice is magnetic resonance imaging. Although radiographic findings usually support diagnosis, the imaging characteristics of such lesions may considerably vary, depending on the histological subtype and the macroscopic appearance of the tumor. The treatment of choice is complete surgical excision when possible. Although the role of postoperative radiotherapy is not clearly defined, some authors support that radiotherapy might delay or prevent local recurrence. However, there is no adequate evidence that the combination of surgery and radiotherapy lowers the possibility of distant metastasis of the head and neck liposarcomas. The role of adjuvant or neoadjuvant chemotherapy still remains controversial.

## 1. Introduction

 Liposarcoma is a quite common soft tissue malignant tumor in adults. In fact, it is second only to fibrosarcoma, as the most frequently occurring soft tissue sarcoma in adulthood [1–3]. The majority of liposarcomas arise in the lower extremities and retroperitoneum, while the incidence of this tumor in the head and neck region is reported to be extremely low, comprising 1.8%–6.2% of all cases [1, 4, 5]. Nasopharyngeal liposarcoma is exceptionally rare, with only three cases having been presented in the English literature [1, 5, 6]. The aim of the present paper is to report a case of a nasopharyngeal liposarcoma, treated with endoscopic tumor debulking, followed by adjuvant chemotherapy and radiotherapy. We also review the current literature, with regard to diagnosis and treatment of such rare malignancies.

## 2. Case Report

**Figure 1:**
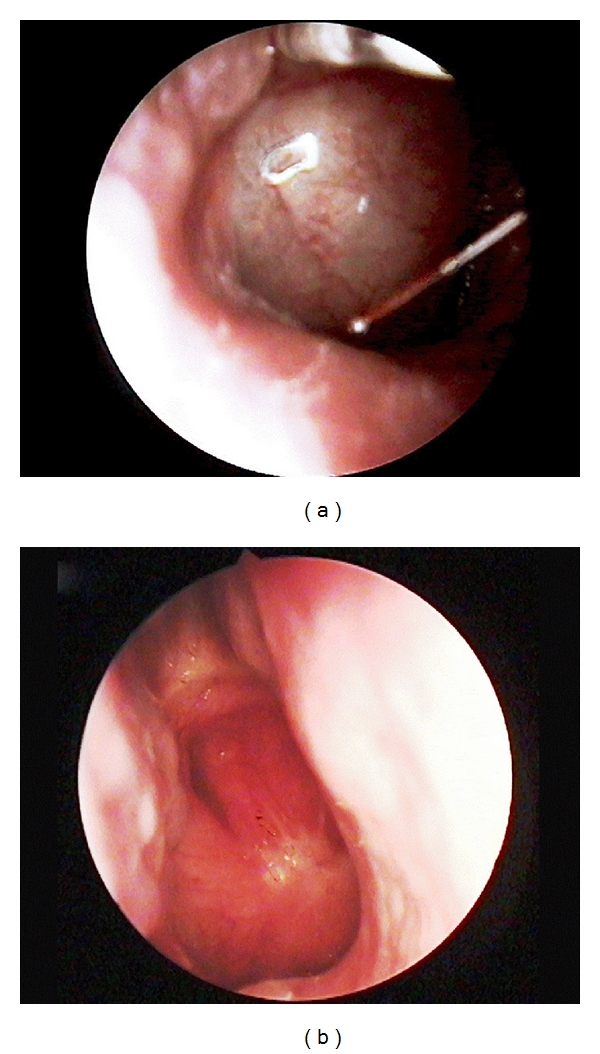
(a) Preoperative endoscopic image of the right-sided tumor situated at the posterior wall of the nasopharynx. (b) Endoscopic follow-up image of the nasopharynx 14 months after postoperative radiotherapy.

**Figure 2:**
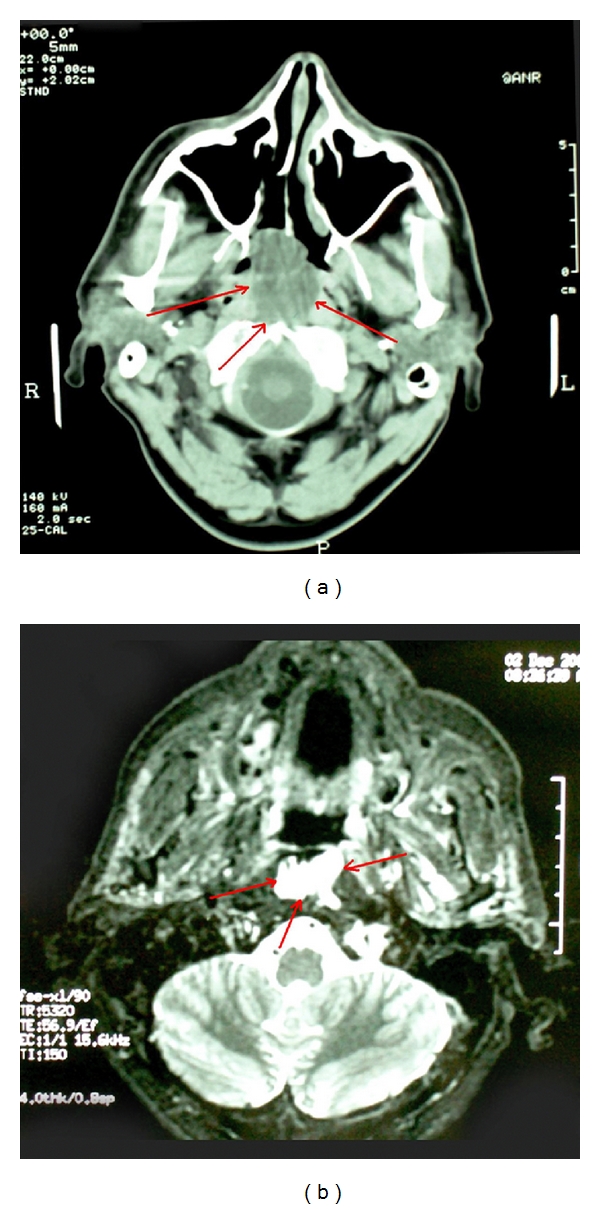
CT (a) and MRI (b) imaging of the tumor (red arrows).

A 58-year-old male presented to our department with a 6-month history of nasal obstruction and right otalgia. Subsequent nasopharyngeal endoscopy revealed a right-sided mass, situated at the posterior wall of the nasopharynx (Figure 1(a)). High-resolution computed tomography (CT), as well as magnetic resonance imaging (MRI), showed a right-sided mass, measuring approximately 2 × 3 × 1.8 cm (Figure 2). There was no imaging evidence of cervical lymphadenopathy.

As the tumor was not possible to be radically excised, our patient underwent an endoscopic biopsy of the mass along with tumor debulking, under general anesthesia.

**Figure 3:**
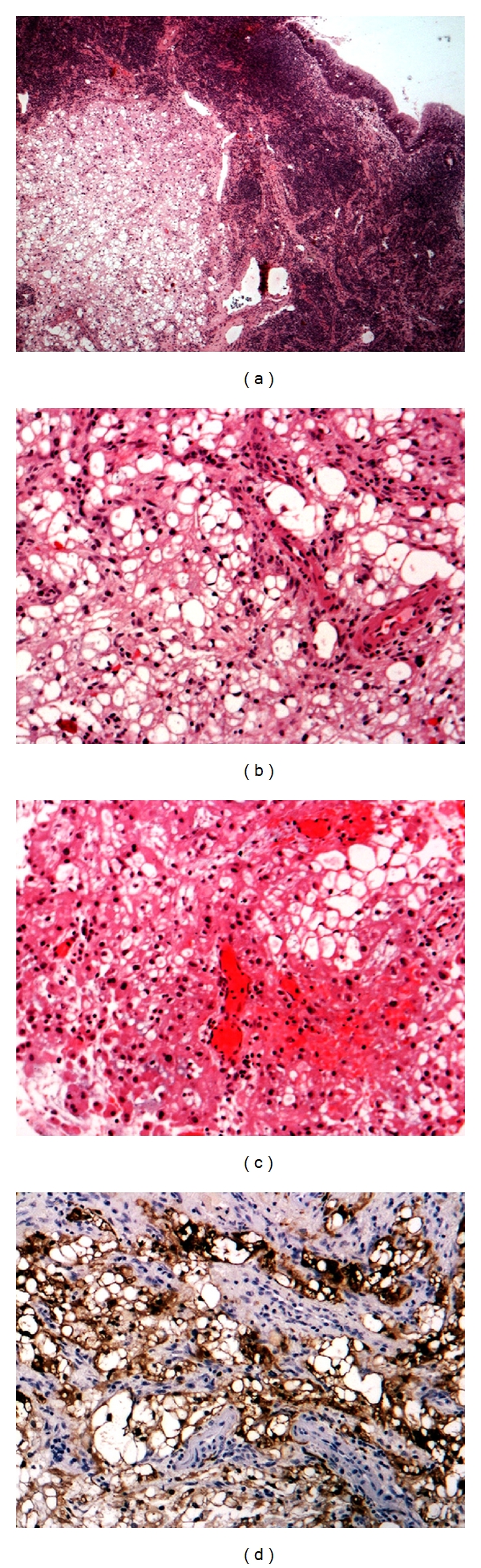
Histopathological images of the presented case. (a) Submucosal (surface epithelium in upper right) liposarcoma (H&E stain, ×25) (b) Myxoid liposarcoma, with lipoblastic differentation (H&E ×100) (c) Liposarcoma with round cells (H&E ×100) (d) Mostly nuclear and some cytoplasmic immunoreactivity for S100 protein in the tumor cells (×100).

The histopathological examination showed a myxoid-round cell liposarcoma, with moderate differentiation and hemorrhage infiltration. Immunoperoxidase stains were positive for S100 protein, vimentin, CD68 in histiocytes, and Ki67 in less than 10% of tumor cells, being negative for SMA, NSE (Figure 3).

Right after the operation, our patient underwent 6 cycles of adjuvant chemotherapy (a doxorubicin-based regimen was administered), followed by radiotherapy (66 Gy in 33 fractions). The patient showed no residual tumor on endoscopy and MRI imaging by the end of radiotherapy. There are still no signs of recurrence on follow-up endoscopy (Figure 1(b)) and MRI imaging, 14 months after radiotherapy.

## 3. Discussion

Liposarcoma is the second most common soft tissue sarcoma, accounting for 14–20% of all soft tissue malignancies [1, 2, 4]. This tumor shows a male preponderance, with the vast majority of the cases diagnosed in adults, between 40 and 60 years of age [2, 7]. Liposarcoma usually arises in the lower extremities and retroperitoneum [1–4]. However, 1.8%–6.2% of these tumors are found in the head and neck region [1, 2, 4, 7]. Nasopharyngeal liposarcoma is exceedingly rare. Only three cases have been described in the English literature [1, 5, 6] (Table 1).

The review of the English literature showed that only a few publications have directly addressed head and neck liposarcomas, usually presenting quite limited patients' series [8]. Based on data and knowledge from the largest reported series of head and neck liposarcomas [8–10], a comprehensive discussion focusing on demographic and clinical/histopathological features, sites of presentation, treatment strategies, and final outcome, in terms of survival, metastasis, and local recurrence rates, was attempted.

The mean age of presentation is between 40 and 46 years, with a peak at the 6th and 7th decades [8–10]. Liposarcomas of the head and neck show a male preponderance (male-to-female ratio is reported to vary between 1.5 and 1.85/1 [8–10]). The neck or parotid region is reported as the most common primary tumor site, followed by skull base/orbit, upper aerodigestive tract (larynx, pharynx, and oral cavity), and scalp or face [8, 9].

Liposarcoma originates from primitive mesenchymal cells. It is divided into five major histologic subtypes: well differentiated, myxoid, dedifferentiated, round-cell, and pleomorphic subtype [1, 2, 4, 11]. The most common histological type is the well-differentiated one (this type includes the adipocytic, sclerosing and inflammatory subtype), accounting for 40–50% of all types, while the most infrequent type is pleomorphic liposarcoma. Myxoid type liposarcoma (similar to the tumor in our case) is composed of three main tissue components, proliferating lipoblasts at varying stages of differentiation (Figure 3(b)), a delicate plexiform capillary pattern, and a myxoid matrix containing abundant nonsulfated glycosaminoglycans [1, 2, 4, 12].

Well-differentiated, myxoid, and pleomorphic types are the most commonly found, while round-cell type lesions are quite rarely confirmed [8–10]. The well-differentiated tumors appear to have excellent prognosis: Golledge et al. reported that none of the patients, diagnosed with such histological subtypes, died during the follow-up period (mean follow-up was 5 years [8]). On the contrary, round-cell and pleomorphic subtypes are usually related with poor prognosis [8–10].

Early diagnosis of liposarcomas is quite rare mainly due to the fact that these tumors are usually painless, gradually developing lesions. Various compressive symptoms may be encountered, depending on the location and the size of the tumor. In the reported cases of nasopharyngeal lesions, as well as in our case, the main symptoms were progressive nasal obstruction along with unilateral persisting otitis media with effusion and referred otalgia [1, 5, 6].

Most authors agree that the imaging modality of choice is MRI [1, 13, 14]. However, computed tomography (CT) is still recommended, especially when data regarding bony or cartilage erosion is required. Although radiographic findings usually support diagnosis, the imaging characteristics of such lesions may considerably vary, depending on the histological subtype and the macroscopic appearance of the tumor [1, 13, 14]. In our case, the myxoid type liposarcoma of the nasopharynx was quite well defined on MRI with homogenous T1 and T2 relaxation times almost similar to those of water. On the other hand, the well-differentiated lesions, which are predominantly fatty, usually show a high signal intensity on T1-weighted sequences and intermediate signal on T2-weighted sequences, with signal attenuation following fat saturation [13, 14].

Provided that nasopharyngeal liposarcomas are exceedingly rare, there are very limited reports in the literature with regard to the management of such malignancies [1, 5, 6]. Most therapeutic protocols and experience are basically based on treatment strategies involved in liposarcomas of the head and neck or other anatomic regions [2–4, 8–10, 15, 16]. 

Liposarcoma is, like other soft tissue sarcomas, primarily a surgical disease. The main goal of surgery is to remove the tumour entirely and prevent recurrence [8–10]. Incomplete resection seems to affect prognosis by increasing the rate of local recurrence or distant metastases in aggressive lesions, such as round-cell or pleomorphic liposarcomas [2, 4, 12].

However, treatment strategies in head and neck liposarcomas are usually significantly different, compared to those related to similar lesions of other regions [8–10]. This fact reflects the limitations caused by anatomical boundaries, as well as expected comorbidity [8, 10]. The site of the tumor, when situated in the head and neck area, might produce serious difficulties with regard to surgical treatment. Radical excision, with sufficient healthy margins, is usually quite difficult to be achieved, mainly due to the specific anatomical relationships of the lesions. Besides, the expected postoperative morbidity is considered to be quite unacceptable in many cases. For example, swallowing or respiration problems might delay patients' recovery or even expose their lives to serious risks. Such functional deficits encountered may vary significantly, depending on the size and location of the individual tumor, and are associated with the removal of tissues adjacent to the tumour (i.e., vessels, muscles, nerves, etc. [10]). Although reconstruction of postresection deficits can, in some instances, be advocated to minimize these effects, this is not always easy to be performed. Therefore, the quality of life of such patients remains seriously affected postoperatively.

In fact, all cases of nasopharyngeal liposarcomas, including the present case, were related with various difficulties, affecting treatment strategy. Management strategy and parameters, as well as final outcome of those four cases, are presented and commented below in detail. We also attempt to indicate how the outcomes of potentially altered treatment in such cases compare to the standard treatment of liposarcomas outside the head and neck region. 

In Knowles and Huggill's case [6] (a 12-year-old boy), a small lesion, about 2 cm in diameter, was palpated just behind the right side of the mandible, along with a few small cervical lymph nodes bilaterally. The nose and throat region was clinically healthy and so was the nasopharynx. A month later, a significantly larger mass, extending from the vault of the nasopharynx down to the oropharynx, was palpated. The mass and the adenoids were only biopsied under general anesthesia, because the tumor was considered to be already unresectable, by the authors. Therefore, the patient was treated with radiotherapy alone. Unfortunately, 10 months later, extended lung metastases were identified. The patient's condition steadily deteriorated, and he finally died, 2 months later.

In Nageris et al.'s case [5] (a 28-year-old woman), the tumor was situated at the lower nasopharyngeal wall. The patient underwent surgery via intraoral approach but the tumor was not possible to be radically excised. Therefore, after histopathological confirmation, the patient was treated with adjuvant postoperative radiotherapy. The authors report that the patients is alive and disease free, eleven years postoperatively.

In Chakraborty et al.'s case [1] (a 37-year-old woman), the tumor was situated at the posterior aspect of the inferior turbinate, adjacent to the eustachian tube orifice. After the initial endoscopic debulking and biopsy was performed, the authors suggested surgical resection plus radiotherapy, as a definite therapy. However, the patient declined surgery. As a result, the case was actually treated with radical radiotherapy alone. Right after radiotherapy, follow-up endoscopy and imaging studies revealed residual tumor. This was also confirmed histopathologically. Surgical resection was again suggested but the patient declined. No details, regarding further treatment or follow-up, are reported by the authors.

In our case (a 58-year-old male), the right-sided tumor was situated at the posterior wall of the nasopharynx. As the lesion was not possible to be radically excised, the patient was treated with endoscopic debulking and adjuvant chemotherapy (a doxorubicin-based regimen) and radiotherapy. The patient is alive, showing no signs of residual tumor, on both follow-up endoscopy and imaging studies, 14 months after radiotherapy.

Obviously, radical tumor excision was not possible in all reported cases of nasopharyngeal liposarcoma, mainly due to anatomical limitations correlated with the site of the lesion [1, 5, 6]. Radiotherapy alone, or as adjuvant treatment, was involved in all cases [1, 5, 6]. In two out of the four cases [5], final outcome was reported to be excellent: the patients were alive and disease-free 11 years and 14 months after initial treatment, respectively.

On the other hand, in most cases of liposarcomas outside the head and neck region, surgery alone or a combined modality approach of preoperative or postoperative radiation therapy is used by most authors [17, 18]. The role of chemotherapy is less well defined [17, 18]. The prognosis depends on several factors, including the patient's age, the size, the histologic grade, the stage of the tumour, and the extent of resection. Factors associated with a poorer prognosis include age older than 60 years, tumours larger than 5 cm, and high-grade histology. While low-grade tumours are usually curable by surgery alone, higher-grade tumours are associated with higher local-treatment failure rates and increased metastatic potential [18]. When feasible, wide margin function-sparing surgical excision is the cornerstone of effective treatment, with the goal of preservation of a functional extremity [17]. This may be facilitated by soft tissue reconstructive surgery. Effective treatment of retroperitoneal sarcomas requires removal of all gross disease, while sparing adjacent viscera not invaded by tumor. The prognosis for patients with high-grade retroperitoneal lesions is less favourable than for patients with tumors of other sites, mainly because of the difficulty in completely resecting these tumours and the limitations placed on high-dose radiation therapy [17, 18].

A recent original study, conducted in the USA, came to an interesting conclusion, regarding the prognosis and the final outcome of liposarcomas in the head and neck region, compared to other anatomical locations: with a median follow-up of 45 months, Smith et al. (2011) [19] concluded that 5-year overall survival rate was similar among extremity (86.6%), trunk (84.6%), and head and neck (88.4%) locations. On the contrary, overall survival rate was statistically worse (77.4%) for retroperitoneal/intra-abdominal cases. Moreover, 5-year disease-specific survival was reported to be similar among extremity (98%), thorax (90%), and head and neck (93.2%) locations but statistically worse for both trunk (92.3%) and retroperitoneal/intra-abdominal cases.

Although surgery with negative margins is very well documented as the treatment of choice for all histopathological subtypes of head and neck liposarcomas, routine regional lymph node dissection is not generally recommended, as node metastases of head and neck liposarcomas are quite rare [8, 10]. Patients with low-grade tumors, such as well-differentiated or myxoid subtypes, can be observed after surgery, provided that the tumors had no local extension, and they were radically excised, with negative margins. Postoperative radiotherapy is advocated by most authors, especially in patients with large tumors, high-grade lesions, local extension and incomplete resection-positive margins [8, 10, 20]. Postoperative adjuvant radiotherapy appears to decrease the rate of local recurrence (from 60% to 40%) but it does not seem to improve the overall survival rate or the rate of metastasis [8–10].

 Although the role of chemotherapy is not clearly defined, some authors support the use of neoadjuvant chemotherapy regimens (doxorubicin and dacarbazine based), in selected patients with high-grade tumours or lesions with complex anatomical subsites, reporting quite satisfactory results, especially in terms of local control of the disease [2, 21].

 In our case, although the liposarcoma proved to be of low-grade, we decided to involve adjuvant chemotherapy along with radiotherapy, because the tumor was quite large and radical excision was not possible to be performed, mainly due to the anatomical location of the mass.

As far as the final outcome is concerned, the study of the literature concludes that both disease-specific and overall survival rates of low-grade head and neck liposarcomas (well-differentiated or myxoid subtypes) are much higher, compared to those in patients with pleomorphic or round-cell subtypes [8–10]. Davis et al. reported a disease-specific survival of 100% for well differentiated and myxoid liposarcomas, versus 60% and 40% for round cell and pleomorphic tumors, respectively [9]. Gritli et al. reported 5-year survival rates of 75% for well-differentiated, 70% for myxoid, 22% for pleomorphic and 0% for round-cell liposarcomas [10]. It is quite commonly accepted that patients treated with surgery alone appear to have significantly better outcome: the 5-year survival rate is reported to be better, compared with that of patients treated with combined therapeutic modalities (5-year survival rates 83% versus 63% [8]). Moreover, Golledge supported that local recurrence is also less common in patients treated with surgery alone [8]. This observation obviously reflects the fact that more aggressive tumors are often treated with combined strategies. However, in the recent study of Davis et al. [9], there were no statistically significant differences between patients treated with surgery alone and those treated with surgery and adjuvant therapies, in terms of both local recurrence and distant metastasis.

Local recurrence is considered to be quite common (reported recurrence rates are between 36% and 63%), especially in younger patients and in more aggressive high-grade liposarcomas or tumors with local extension into surrounding tissues [8–10]. Such patients should be selected for more aggressive surgical treatment or even combined surgery and adjuvant radiotherapy. Distant metastases (bony, hepatic, adrenal, and pulmonary) are quite rarely identified by the time of initial diagnosis and are mostly found in women and more aggressive high-grade/larger tumors (>5 cm), usually treated with combined therapeutic modalities (these differences are not documented as statistically significant [8, 9]).

 The site of the lesion also appears to affect prognosis: scalp, facial, and laryngeal liposarcomas are reported to have a relatively better prognosis, probably due to earlier diagnosis, compared with intraoral, pharyngeal, or neck tumors [8].

 In our case, although the lesion was <5 cm in diameter and the histological subtype was of low grade, radical excision was not possible. We decided to use both adjuvant chemotherapy and radiotherapy because of the location of the tumor, which could make the identification of local recurrence very difficult, especially without the use of biopsies. The outcome is very satisfactory, as the patient is alive and disease-free (no signs of local recurrence, regional node, or distant metastasis), 14 months after radiotherapy.

## 4. Conclusions

Liposarcoma is a soft tissue malignant tumor rarely occurring in the head and neck region. Nasopharyngeal liposarcomas are even more rarely diagnosed, with only three cases having been reported in the English literature. Treatment of choice is surgical excision with the wider possible margins, followed by postoperative radiotherapy and adjuvant chemotherapy in selected cases.

## Figures and Tables

**Table 1:** Cases of nasopharyngeal liposarcoma, reported in the English literature.

Case no.	Study	Patient sex and age (years)	Histological type	Treatment	Follow-up	Patient's status by the end of follow-up
1	Knowles and Huggill	Male 12	Not clearly classified	Radiotherapy	10 months	Dead
2	Nageris et al.	Female 28	Myxoid stroma, tumour cells with pleomorphic nuclei	Incomplete excision plus postoperative radiotherapy	11 years	Alive, Disease-free
3	Chakraborty et al.	Female 37	Well-differentiated, sclerosing subtype	Debulking-biopsy followed by radical radiotherapy and stereotactic radiotherapy	Not clearly defined	Residual tumor existed after treatment
4	Present case 2011	Male 58	Myxoid type	Endoscopic debulking, adjuvant chemotherapy, and radiotherapy	14 months	Alive, disease-free
